# Effect of C reactive protein point-of-care testing on antibiotic prescribing for lower respiratory tract infections in nursing home residents: cluster randomised controlled trial

**DOI:** 10.1136/bmj.n2894

**Published:** 2021-11-29

In this paper by Boere and colleagues (*BMJ* 2021;374:n2198, doi:10.1136/bmj.n2198, published 21 September 2021), in figure 1, for the last category (100-200mg/L) the correct number is N=24 for antibiotic start=yes. This has since been rectified, and the article and PDF will be updated in due course.

